# Homomorphic Filtering for Improving Time Synchronization in Wireless Networks

**DOI:** 10.3390/s17040909

**Published:** 2017-04-20

**Authors:** José María Castillo-Secilla, José Manuel Palomares, Fernando León, Joaquín Olivares

**Affiliations:** 1 Department of Computer Technology, University of Alicante, Carretera San Vicente del Raspeig, S/N, 03690 Alicante, Spain; 2 Department of Computer Architecture, Electronics and Electronic Technology, Universidad de Córdoba, Campus de Rabanales, 14001 Córdoba, Spain; jmpalomares@uco.es (J.M.P.); olivares@uco.es (J.O.); 3 PRINIA Research Group, Universidad de Córdoba, Campus de Rabanales, 14001 Córdoba, Spain; fernando.leon@uco.es

**Keywords:** clock skew, WSN, synchronization, temperature, oscillators, tuning-fork, homomorphic filtering, TelosB, 802.15.4, TinyOS

## Abstract

Wireless sensor networks are used to sample the environment in a distributed way. Therefore, it is mandatory for all of the measurements to be tightly synchronized in order to guarantee that every sensor is sampling the environment at the exact same instant of time. The synchronization drift gets bigger in environments suffering from temperature variations. Thus, this work is focused on improving time synchronization under deployments with temperature variations. The working hypothesis demonstrated in this work is that the clock skew of two nodes (the ratio of the real frequencies of the oscillators) is composed of a multiplicative combination of two main components: the clock skew due to the variations between the cut of the crystal of each oscillator and the clock skew due to the different temperatures affecting the nodes. By applying a nonlinear filtering, the homomorphic filtering, both components are separated in an effective way. A correction factor based on temperature, which can be applied to any synchronization protocol, is proposed. For testing it, an improvement of the FTSP synchronization protocol has been developed and physically tested under temperature variation scenarios using TelosB motes flashed with the IEEE 802.15.4 implementation supplied by TinyOS.

## 1. Introduction

The rapid growth of Wireless Sensor Networks (WSN) is leading to numerous research projects [1]. Their increase involves the development of wireless embedded devices, communication technologies and distributed computing. This work focuses on the challenge represented by distributed sensors, which triggers the need for synchronization schemes among the embedded devices that form the entire network.

Real-time applications are one of the main areas of interest in WSN, making necessary the use of accurate coordination mechanisms to guarantee synchronization constraints. One specific field of application is Wireless Multimedia Sensor Networks (WMSNs) [2]. The availability of low-cost hardware, such as cameras and microphones, has fostered the range of applications suitable to use in WMSNs. Among the concept of WMSNs emerge the Visual Sensor Networks (VSNs). A VSN is a collaborative wirelessly interconnected network that retrieves visual information from an environment. As Soro et al. state [3], to manage that visual information, real-time performance and time synchronization are a couple of compulsory requirements for the correct performance of VSN. WSNs suppose an important application field inside the concept of the Internet of Things (IoT) [4], which has become a global communication concept among all kinds of devices capable of connecting to the Internet. Several IoT applications, e.g., data acquisition [5,6] or data fusion [7], are time-dependent tasks and need time synchronization protocols in order to work correctly. Furthermore, as Li et al. [8] state, due to a large number of nodes in complex IoT systems, devices should be designed to minimize cost, size, resource and energy consumption. In the concrete case of energy consumption, time synchronization is essential for optimizing it when nodes are sending or receiving information among themselves.

The above mentioned real-time performance and time synchronization are reached by using time synchronization protocols, e.g., the Flooding Time Synchronization Protocol (FTSP) [9], which is one of the most used synchronization protocols in WSN. Nevertheless, other mechanisms with better accuracy under certain situations are necessary to achieve real-time performance, such as the pseudo-synchronous algorithm [10], PISync [11] or the distributed synchronization algorithms proposed by Luo et al. [12] and Du et al. [13], among others proposals.

In the pseudo-synchronous algorithm, unlike other synchronization protocols, transmissions are not performed synchronously. The simulated results of this proposal outperform significantly when compared to other synchronization strategies in terms of robustness to process, measurement noises and time-varying lock drifts under certain assumptions: (i) initial times between nodes are relatively close to each other; and (ii) the absence of process noise, measurement noise or propagation delays.

PISync synchronizes each sensor of the network by applying a Proportional feedback (P) and an Integral feedback (I) to the synchronization error with respect to the received reference time. This mechanism permits compensating both clock offset and clock skew.

In the distributed clock skew and offset estimation in wireless sensor networks asynchronous algorithm and convergence analysis proposed by Luo et al. [12], each node can estimate its clock skew and offset in a distributed and asynchronous way without requiring any centralized information, processing or coordination.

The proposal of Luo et al. [12], distributed clock parameters tracking in wireless sensor network, develops a distributed Kalman filter for clock parameter tracking. This solution only requires each node to exchange limited information with its neighbors, which improves energy consumption and scalability.

The clock skew appears in every mote, and it is determined by the ratio between the actual oscillator frequency in each of them. This is due because every mote showing minimal differences between their XO (Crystal Oscillator), even for the same model of oscillator. It is known that oscillators are highly influenced by temperature [14,15], and as a consequence, the clock skew varies when temperature changes. In order to avoid this influence, several different approaches have been taken. The first approach is to produce oscillators that compensate the effects of temperature, such as TCXO (Temperature-Compensated Crystal Oscillator), or that avoid temperature changes by a very efficient encapsulation, OCXO (Oven-Controlled Crystal Oscillator). These are very precise, although they are very expensive. Other approaches are the design of new time synchronization protocols with temperature compensation. Among these new time synchronization protocols, the following can be highlighted: TCTS [16], Virtual High-resolution Time (VHT) [17], Temperature-Aware Compensation for Time Synchronization (TACO) [18] and Environment-aware clock skew estimation and synchronization (EACS) [19].

Another option is to include a temperature compensation factor [20] within a time synchronization clock-skew-based protocol, for instance, FTSP. This approach was suggested in Adjusted Temperature FTSP (AT–FTSP) and Advanced Adjusted Temperature FTSP (A2T–FTSP) [14]. In that work, clock skew was compensated with a factor taking into account temperature. Those mechanisms showed their effectiveness and accuracy [14,15] with low latency and overhead.

Analyzing those results, a hypothesis can be stated: frequency (and therefore, clock skew) is mainly composed of two multiplicative components: one due to the cut of the crystal and the other due to the temperature. Thus, if the component due to temperature is removed from the clock skew and substituted by the temperature compensation factor introduced in the AT and A2T mechanisms, much better accuracy is obtained. In order to separate those multiplicatively combined components, a non-linear filtering has to be applied. In this work, a homomorphic filtering [21] is used to separate the multiplicatively-combined components of the clock skew.

The separation of the components allows one to use the clock skew due to the cut of the crystal of the oscillators (called $skew_{\mathrm{cut}}$) and the one due to temperature (called $skew_{\mathrm{temp}}$). Applying a correction factor to the $skew_{\mathrm{cut}}$, a more accurate synchronization can be achieved. FTSP [9] has been selected as the main time synchronization protocol, obtaining a couple of new filtered time synchronization protocols, called HF–FTSP (Homomorphic Filtered FTSP) and HF2–FTSP (Homomorphic Improved Filtered FTSP). Experimental results have been obtained in a network of TelosB (Rev. b) [22] motes running TinyOS [23] with the IEEE 802.15.4 [24] communication stack. The results show that HF2–FTSP improves the average synchronization error with respect to FTSP and other advanced proposals (A2T–FTSP [14,15,25]).

### Mathematical Nomenclature

In this work, some mathematical terms will be used, and they are going to be introduced first. Please refer to each section for further information on how each term is used and managed. Table 1 describes the mathematical terms.

## 2. Multiplicative Combination of Components Involved in Clock Skew

A multiplicative signal is composed by a couple of signals, which as stated by Oppenheim [21,26], can be separated. Based on the work of Vig [27], there are multiple factors involved in the real frequency of the oscillators. Some of them change slowly with time (e.g., cut of crystal, aging, etc.), while others show a faster response on the oscillation frequency (e.g., temperature, accelerations, magnetic fields, atmospheric pressure, voltage, etc.). Thus, the cut of the crystal and aging could be seen as low frequency signals because of their low rate of changing. Moreover, the other factors that make the oscillation change quickly can be classified into high frequency signals.

Therefore, as clock skew is a relationship between frequencies, the main hypothesis of this work is that clock skew is formed by several components that change with time. These components are the clock skew due to the cut of the crystal and a dynamic clock skew characterized by certain changing conditions. Among these dynamic conditions, temperature affects more heavily and directly the final clock skew value [15]. For this reason, the clock skew estimation for an instant time in a WSN mote can be seen as a combination formed by a low frequency component (skew due to cut of the crystal oscillator) and a high frequency component (skew due to temperature).

Following the previous work of Castillo et al. [25], the main parameter that makes the value of the high frequency clock skew vary is the temperature in the crystal oscillator. An immediate change in the temperature will change the resonance frequency of the oscillator, and as a consequence, the clock skew will also change. For this reason, temperature is a critical parameter in the clock skew estimation. Based on the above-mentioned hypothesis, a more precise clock skew value could be obtained through the separation of the low frequency component and the high frequency components. It is possible to make the same assumption that Oppenheim [21] did, considering that the combination of those signals is made by means of a product.

As Equation (1) states, the final signal is the multiplicative result of the combination of the low and high frequency clock skew, and thus, it can be decomposed by using homomorphic filtering of multiplied signals, where $skew_{\mathrm{cut}}$ is the skew due to the cut of the crystal oscillator and $skew_{\mathrm{temp}}$ is the one due to temperature effects:

(1) $$\mathrm{skew}\left(t,\vec{T}\right)=skew_{\mathrm{cut}}\left(t\right)\cdot skew_{\mathrm{temp}}\left(t,\vec{T}\right)$$

These variables have been introduced in Section 1. However, some further explanations are provided here. The instantaneous skew (either the complete skew, skew, or the skew components, $skew_{\mathrm{cut}}$ or $skew_{\mathrm{temp}}$) at a given time is denoted by *t*, and $\vec{T}$ represents the evolution of the temperature, considered as a signal or, mathematically, as a vector.

## 3. Homomorphic Filtering for the Separation of Components Involved in Clock Skew

This section states how to improve the efficiency of the synchronization through the separation of the two main components that affect the clock skew. The clock skew at each instant is computed using a linear regression model of current and several past clock values fitted with a least squares approach. The series of clock skew figures can be treated as a signal of temporally-ordered values. Thus, signal processing methods can be applied to it. In particular, the skew signal can be processed to be separated into its core components.

The separation of components is represented in Equation (1), where $skew_{\mathrm{cut}}\left(t\right)$ is the skew due to the low frequency components (mainly due to the cut of the crystal) and $skew_{\mathrm{temp}}\left(t,\vec{T}\right)$ represents the higher frequency components (mainly due to temperature effects). The $skew_{\mathrm{cut}}\left(t\right)$ depends only on the time *t*, whilst $skew_{\mathrm{temp}}\left(t,\vec{T}\right)$ depends on both the instant time (*t*) when requested and the evolution of temperature (the vector of temperatures, $\vec{T}$). For simplicity, the variable arguments of the components will not be included in the rest of the article. For instance, $skew_{\mathrm{cut}}\left(t\right)$ will be denoted $skew_{\mathrm{cut}}$.

Oppenheim et al. [21] described the separation of the high and low frequency components of a multiplicative signal applying filters in a logarithmic space. Therefore, Equation (1) can be processed using the method proposed by Oppenheim et al., providing Equation (2).

(2) $$\ln\left(\mathrm{skew}\right)=\ln\left(skew_{\mathrm{cut}}\right)+\ln\left(skew_{\mathrm{temp}}\right)$$

It is possible to obtain the lower frequencies of the skew signal series by applying a Low-Pass Filter (LPF) to the logarithmic transformation of the clock skew series treated as a signal. Thus, as a first estimation, it is possible to obtain an approximation of the $\ln(skew_{\mathrm{cut}})$ as shown in Equation (3). Some considerations on the selection of the LPF will be introduced in Section 3.1.

(3) $$\ln\left(skew_{\mathrm{cut}}\right)\approx LPF\left(\ln(\mathrm{skew})\right)=\ln\left(LPF(\mathrm{skew})\right)$$

The moving average filter, Avg(·), is a function that provides an LPF behavior. Thus, the average value is considered a representation of the low frequency components of the skew in the logarithmic space. Therefore, let $\mathrm{Avg}\left(skew\right)=skew_{\mathrm{Avg}}$, so it is possible to use the first order approach to the value of the ${\mathrm{skew}}_{\mathrm{cut}}$, stated in Equation (4).

(4) $$\ln\left(skew_{\mathrm{cut}}\right)\approx \ln\left(skew_{\mathrm{Avg}}\right)$$

Using Equations (2) and (4), Equation (5) is obtained.

(5) $$\ln\left(\mathrm{skew}\right)=\ln\left(skew_{\mathrm{Avg}}\right)+\ln\left(skew_{\mathrm{temp}}\right)$$

These expressions permit one to obtain a clock skew value based on temperature as shown in Equation (6).

(6) $$skew_{\mathrm{temp}}=e^{\ln\left(\mathrm{skew}\right)-\ln\left(skew_{\mathrm{Avg}}\right)}$$

### 3.1. Considerations on the Low-Pass Filter

Some considerations have to be taken into account in order to use an Low-Pass Filter (LPF) that is able to discard properly the high frequency components. First of all, the filter should be able to eliminate (or at least to largely attenuate) the frequencies that are not related to the nominal frequency of the oscillator. For most commercial non-compensated oscillators, the frequency tolerances are typically in the range of ±10–100 ppm. Therefore, two XOs working at stable ambient temperature would oscillate at their nominal frequency, and their ratio of frequencies (skew) would be constant. However, due to the frequency tolerance, some random walks in the actual frequency of the XOs may occur, and thus, the skew could change slightly from that constant value mentioned above. However, these random walks are constrained by the frequency tolerance, and it is rather improbable to get frequencies outside the tolerance range.

If the single-sided amplitude spectrum of the discrete Fourier transform is applied on the vector composed by a temporal series of skew, a large amplitude peak would appear at 0 Hz, which represents the skew of the XOs working at their nominal frequency. The peak drops quickly as moving away to higher frequencies in the spectrum. The frequency of that spectrum at which the amplitude changes the slope provides the cut-off frequency for low-pass filtering. The random walks for the tolerance frequency of the oscillators lay in high frequencies, but the amplitudes of those peaks are really small, and therefore, negligible.

On the other hand, changes in temperature affect quickly the frequency of the oscillators; thus, some peaks appear outside the low frequencies band defined by the cut-off frequency described before.

The Moving Average Filter (MAF) [28] is optimal for reducing random noise while keeping a sharp step response. It may be used as an LPF, although it shows a slow roll-off and limited capacity of band attenuation. In spite of these bad features, it is widely used because of its simplicity, which makes it one of the few LPF available in highly-constrained computational devices.

The ripple of the skew signal has to be mitigated by the MAF to work properly as an LPF. Therefore, the width of the filter has to be long enough to cope with the dynamic impact of the temperature effect. Several features have a direct impact in it: the amplitude and quickness of the change in temperature, the stability of the temperature during long periods, hysteresis, etc. It is out of the scope of this work to analyze thoroughly each feature and its impact in the width of the MAF to obtain a stable $skew_{\mathrm{cut}}$ value.

When the MAF is used as an LPF, the cut-off frequency ($F_{c}$) depends on the used window size according to Equation (7), where *N* is the number of elements of the window and $T_{s}$ is the lapse of each element in seconds (in this case, the synchronization beacon time rate). Thus, it is necessary to analyze the temperature changes in order to select the cut-off frequency. Obviously, this fact depends on each experiment.

(7) $$F_{c}=\frac{1}{\left(N\cdot T_{s}\right)}$$

The cut-off frequency may be complex to select directly, so it is easy to process the signal in the time domain. Several experiments with real conditions have shown that the width of the MAF, that is, the $N\cdot T_{s}$ value in Equation (7), should be, at least, twice as wide as the temporal window of the temperature change. Narrower filters will not be able to attenuate temperature changes, and therefore, they would provide a filtered version of the original skew, which is useless for the purposes of this work. Wider filters would provide more stable behaviors, at the expense of longer periods and larger computational processing. An example of these considerations for a real experiment will be shown in Section 4.2.

### 3.2. Obtaining a Clock Skew Estimation with FTSP

FTSP is a widely-used time synchronization protocol that provides good results in terms of clock skew estimation. As Maróti et al. [9] state, the FTSP time-stamping protocol reduces effectively all sources of time-stamping errors except for the propagation time. The estimation of the clock skew due to temperature may be afflicted by the additive behavior of the message jitter, which could lead to errors in the computation of the high frequency signal separation of the $skew_{\mathrm{temp}}$. The purpose of this work is to demonstrate that it is possible to separate the main components involved in clock skew, and for this reason, it is necessary to remove the uncertainties due to message jitter. Therefore, in order to reduce the communication jitter that FTSP includes, an approach has been used in which the root node time is excluded from the computation of the time synchronization error. It is straightforward to assume that the broadcast beacon reaches every node at the same time because the propagation delay is considered to be less than 1 μs for up to 300 m [9]. In our experimental setup, all nodes are relatively close (between 5 m and 15 m) among each other, and this is why we can assume that propagation delay is small enough for considering it almost negligible in our experiments.

For the sake of the comparison with other approaches, FTSP has been selected as the base protocol to demonstrate the hypothesis stated in this work. Having in mind the previous paragraph and using the previous clock skew equations applied to FTSP, a new synchronization method called HF–FTSP is proposed. Besides, it is possible to incorporate more information for clock skew if the temperature in the main clock is known. In that case, a new alternative method called HF2–FTSP is proposed.

### 3.3. Tuning-Fork Crystal Oscillators

Tuning-fork oscillators are characterized by a quadratic relationship between their resonance frequency and the temperature. Based on that assumption, the previous work of Castillo et al. [15] proposes two temperature-based correction factors to improve the clock skew estimation for temperature variations. Including the actual temperature of the motes in the computations, it is possible to obtain a correction factor that minimizes the effects of the temperature in the oscillators frequency.

The first approach proposed in that work was called AT–FTSP. Based on the quadratic relationship of tuning-fork oscillators, in [29], it is demonstrated that it is possible to obtain a correction factor of the clock skew using the temperature of the local mote (See Equations (8) and (9)).

(8) $$correctionFactor_{\mathrm{simple}}=1+\beta_{n}{\left(\Delta T_{n}\right)}^{2}$$

(9) $$\mathrm{skew}=skew_{\mathrm{FTSP}}\cdot correctionFactor_{\mathrm{simple}}$$

where $\beta_{n}$ and $T_{n}$ are, respectively, the temperature coefficient and the current temperature of the node to be compensated. In the oscillator used in this work (CMR200T) β=−0.034±0.006 ppm, and the frequency tolerance is about ±20 ppm. This working interval is mainly due to the effects of the cut of the crystal.

The second approach proposed in [29] is A2T–FTSP. If the temperature in the root mote is known, a more precise clock skew in the local mote can be obtained (see Equations (10) and (11)). The only requirement is to have temperature sensors both in the root and the local motes. Let $\beta_{r}$ and $T_{r}$ be, respectively, the temperature coefficient and the current temperature of the root node.

(10) $$correctionFactor_{\mathrm{extended}}=\frac{1+\beta_{n}\Delta T_{n}^{2}}{1+\beta_{r}\Delta T_{r}^{2}}$$

(11) $$\mathrm{skew}=skew_{\mathrm{FTSP}}\cdot correctionFactor_{\mathrm{extended}}$$

For the sake of simplification of the computation, if the same type of oscillator is used, it can be considered $\beta_{n}=\beta_{r}$.

The proposed correction factors have been used to add into the $skew_{\mathrm{cut}}$ multiplicative component due to temperature effects.

### 3.4. HF–FTSP

If the temperature in the main clock of the WSN cannot be obtained or it is fixed to the nominal working temperature of the oscillator, it can be excluded from the clock skew estimation, decreasing the amount of computation in the CPU of the mote. To achieve that, a clock skew estimation based on the AT–FTSP synchronization protocol is proposed:

(12) $$skew_{\mathrm{HF}-\mathrm{FTSP}}=skew_{\mathrm{cut}}\cdot correctionFactor_{\mathrm{simple}}$$

### 3.5. HF2–FTSP

If the temperature in the main clock is known, it is interesting to include it in the clock skew estimation in order to improve the accuracy. In this case, a similar temperature adjustment as proposed in A2T–FTSP can be stated in Equation (13).

(13) $$skew_{\mathrm{HF}2-\mathrm{FTSP}}=skew_{\mathrm{cut}}\cdot correctionFactor_{extended}$$

The result of the mathematical postulation can be seen in the pseudocode for HF–FTSP (see Algorithm 1), as well as HF2–FTSP (see Algorithm 2). Furthermore, Table 1 summarizes all of the variables used in the mathematical model proposed.

**Algorithm 1** Skew for HF–FTSP.1:**procedure** HF-FTSP2:   skewSum←03:   **for** (i < 4) **do**4:   skewSum←skewSum+tableSkew[i].skew5:   **end for**6:   newSkewAverage←skewSum/tableEntriesSkew7:   skewT←exp(ln(newSkew)−ln(newSkewAverage))8:   skewCut←newSkew/skewT9:   $correctionFactor_{\mathrm{simple}}\leftarrow 1+\left(\left(BETA/1E6\right)\cdot {\left(temperature-25\right)}^{2}\right)$10:   $skew_{\mathrm{HF}}-\mathrm{FTSP}\leftarrow skewCut\cdot correctionFactor_{\mathrm{simple}}$   **return** $skew_{\mathrm{HF}}-\mathrm{FTSP}$11:**end procedure**

**Algorithm 2** Skew for HF2–FTSP.1:**procedure** HF2-FTSP2:   skewSum←03:   **for** (i < 4) **do**4:   skewSum←skewSum+tableSkew[i].skew5:   **end for**6:   newSkewAverage←skewSum/tableEntriesSkew7:   skewT←exp(ln(newSkew)−ln(newSkewAverage))8:   skewCut←newSkew/skewT9:   $nodeCorrection\leftarrow 1+(\left(BETA/1E6\right)\cdot {\left(temperature-25\right)}^{2})$10:   $rootCorrection\leftarrow 1+(\left(BETA/1E6\right)\cdot {\left(rootTemperature-25\right)}^{2})$11:   $correctionFactor_{\mathrm{extended}}\leftarrow nodeCorrection/rootCorrection$12:   $skew_{\mathrm{HF}}2-\mathrm{FTSP}\leftarrow skewCut\cdot correctionFactor_{\mathrm{extended}}$   **return** $skew_{\mathrm{HF}}2-\mathrm{FTSP}$13:**end procedure**

## 4. Hypothesis Demonstration

This section is focused on the demonstration of the hypothesis stated in Section 3. For that, experiments with and without temperature variations have been executed to demonstrate the existence of the low frequency and high frequency components in the clock skew.

**Figure 1:**
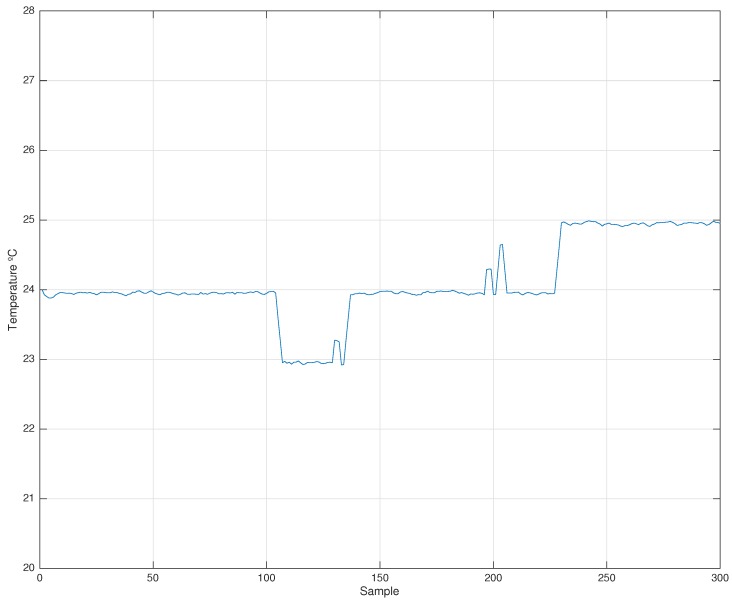
Measured temperature at Node #2.

The demonstration has been carried out under two different execution scenarios. The parameters of the testbed for both experiments are shown in Table 2. Nevertheless, the first one has been performed in a temperature-fixed scenario (see Figure 1) and the second one with a large temperature change (see Figure 3).

**Figure 2:**
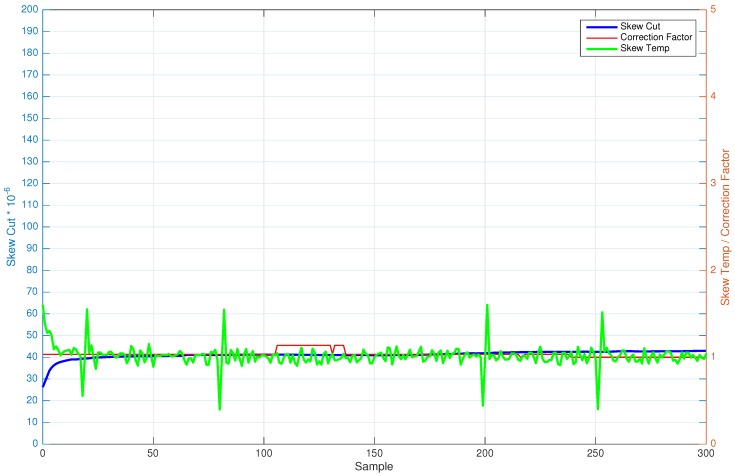
Comparative between skew cut, skew tempand correction factor without temperature variation.

In both scenarios (see Figure 2 and Figure 6), the two extracted components ($skew_{\mathrm{cut}}$ and $skew_{\mathrm{temp}}$), as well as the correctionFactor are shown. Besides, Table 3 and Table 4 show numerically the relationship of $skew_{\mathrm{cut}}$ and $skew_{\mathrm{temp}}$ for two different nodes of the deployed network.

### 4.1. Experiment without Temperature Variation

A test without temperature variation has been carried out in a real scenario under ambient temperature during almost 50 minutes (3000 s), which corresponds to about 300 synchronization points. Figure 2 shows the average clock $skew_{\mathrm{cut}}$, $skew_{\mathrm{temp}}$ and correctionFactor of Node 2. As can be seen, the $skew_{\mathrm{cut}}$ remains fixed during the entire time of execution, while the $skew_{\mathrm{temp}}$ suffers slight variations across time.

Table 4 shows the skew values obtained in certain times of Node 2. As can be seen, $skew_{\mathrm{cut}}$ is almost constant (except for the above-mentioned slight variations), while the $skew_{\mathrm{temp}}$ is oscillating its value with time. This behavior demonstrates the initial hypothesis: “the clock skew estimation for an instant of time in a WSN mote can be seen as a multiplicative combination formed by a low frequency component (skew due to cut of the crystal oscillator) and a high frequency component (skew due to temperature)”. In order to enforce the assumption, Table 4 has a column with the skew value obtained with FTSP ($skew_{\mathrm{FTSP}}$). Comparing this value with the multiplication of the two obtained values ($skew_{\mathrm{cut}}\cdot skew_{\mathrm{temp}}$), the results are basically the same, demonstrating completely the initial hypothesis.

### 4.2. Experiment with Temperature Variation

**Figure 3:**
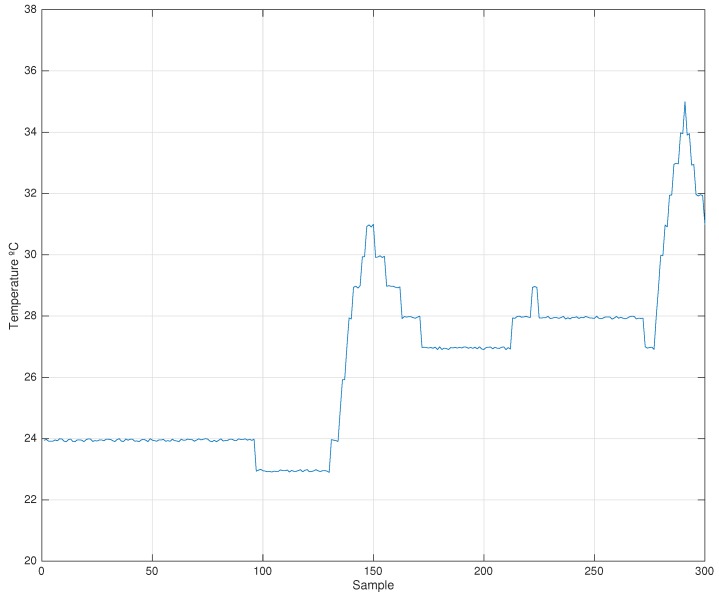
Temperature for the real-world experiment.

This section demonstrates that skew is composed of high and low frequency signals combined in a multiplicative relationship between them in a temperature-variable scenario. The low frequency signals are mainly represented by the cut of the crystal oscillator while, on the opposite, it can be considered that temperature variations affect high frequency signals. For demonstrating this hypothesis a 3000-s experiment has been done with a synchronization rate of 10 s, which supposes about 300 synchronization points. During the experiment, temperature changes several times, as can be seen in Figure 3.

#### 4.2.1. Selecting the Proper Low-Pass Filter Configuration

As has been described in Section 3.1, experiments have shown that for changes in temperature in real-life environments (for instance, with temperature gradients of $0.2{\;}^{\circ }$C/segin a $6^{\circ }$C–$8^{\circ }$C change), the width of the moving average filter should be, at least, twice as wide as the temporal window of the temperature change.

**Figure 4:**
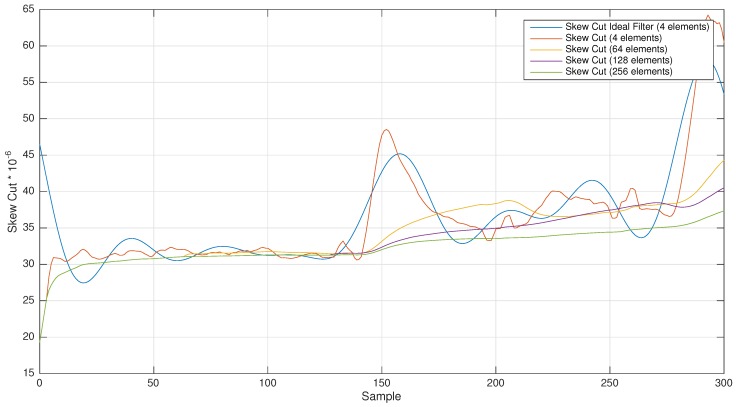
Comparative skew cut using different window sizes (ranging from 4–256).

**Figure 5:**
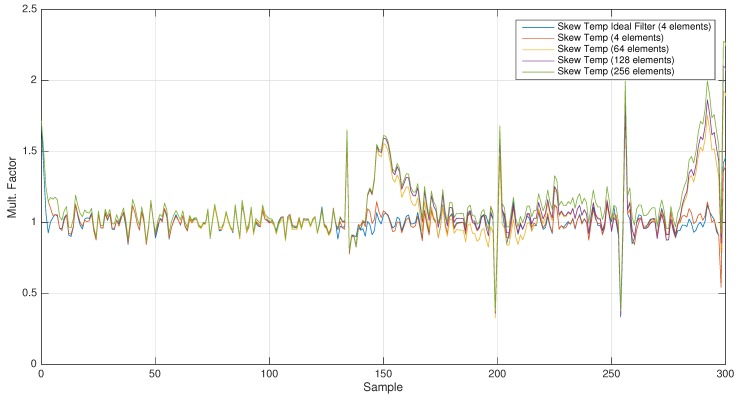
Comparative skew temp obtained from skew cut using different window sizes (ranging from 4–256).

In Figure 3, the temperature of Node 4 is shown. The reference node (root node) is set at $25{\;}^{\circ }$C with no change in temperature. Figure 4 and Figure 5 show different $skew_{\mathrm{cut}}$ and $skew_{\mathrm{temp}}$ using different window sizes of the filter. It can be shown that the $skew_{\mathrm{cut}}$ takes an almost stationary behavior with sizes bigger than 64 elements (it is to be noted that each element is a synchronization beacon with a 10-s beacon rate). There is a change in temperature (from $24{\;}^{\circ }$C–$29{\;}^{\circ }$C) from 1300–1550 (250 s), from base to peak. Therefore, at least a filter with a 640-s width is necessary. With this size, the filter is able to include more values from the stable temperature stage, and therefore, it is able to mitigate the temperature peak. With fewer values, the average filter is not able to reduce it enough. Temperature changes with slower gradient or narrower amplitude can be dealt with by the linear regression procedure, and therefore, the skew is modified accordingly, providing a very good approximation with a small synchronization error. In those cases, the $skew_{\mathrm{temp}}$ is small, and small changes in the $skew_{\mathrm{cut}}$ and the offset are able to cope properly with the temperature changes.

Based on the results obtained in the above-mentioned testbed, several different LPF configurations have been chosen for filtering the skew signal.

For demonstrating our hypothesis, an MAF has been selected with a variable window size of 4, 64, 128 and 256 elements (with cut-off frequencies of 0.025 Hz, 0.001 Hz, 0.0007 Hz and 0.0003 Hz, respectively). The obtained results show how this filter works, separating the low frequency signals (see Figure 4 and Figure 5).

#### 4.2.2. Analyzing the Obtained Results

**Figure 6:**
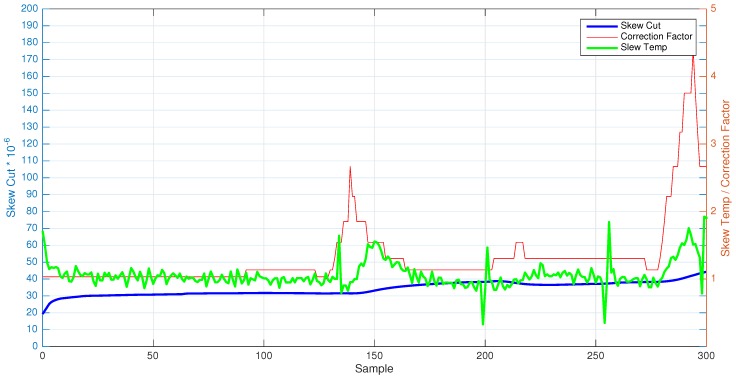
Comparative between skew cut, skew temp and correction factor with temperature variation.

Figure 6 shows both temperature and average clock skew of the nodes in the network. As can be seen once again, the value of the clock skew remains practically stable during the entire test. On the other hand, the $skew_{\mathrm{temp}}$ suffers from the temperature variation almost immediately when this one changes. These results state the hypothesis about the separation of components in the clock skew. To seek the comparison with the temperature-fixed experiment, Table 4 shows the clock skew value for a node in which the temperature variation has been applied. As can be seen in Table 4, $skew_{\mathrm{cut}}$ remains almost stable, while $skew_{\mathrm{temp}}$ is highly affected when temperature changes. If these values are multiplied between them ($skew_{\mathrm{cut}}\cdot skew_{\mathrm{temp}}$), the obtained results are the same compared to the clock skew obtained with FTSP ($skew_{\mathrm{FTSP}}$) except for the minimal floating-point differences (The DIFF SKEW column in Table 4).

### 4.3. Analyzing the Quadratic Relationship between the Temperature and the Skew due to Temperature in Tuning-Fork Oscillators

In tuning-fork oscillators, the effective frequency has a quadratic relationship with respect to the temperature. On the other hand, skew is the ratio between two frequencies, and thus, the relationship between the ratio of frequencies and the temperature is not directly observable and may not show directly a quadratic behavior.

The following lines are focused on analyzing this behavior using the analytical data shown in Table 4. These values are sorted and grouped on the temperature (see Table 5); thus, they are temperature-ordered (and grouped) and not timed-ordered. This sorting allows for a much clearer representation of the $skew_{\mathrm{temp}}$ behavior. The values of $skew_{\mathrm{temp}}$ shown are computed assuming that the reference oscillator remains stabilized at $25^{\circ }$ (thus, it does not suffer from any temperature influence). The expected $skew_{\mathrm{temp}}$ is obtained using Equation (14), which can be derived from the quadratic relationship formula of the tuning-fork [15].

(14) $$ExpectedSkew_{\mathrm{temp}}=1+\beta \cdot {\left(T-25^{\circ }\right)}^{2}$$

**Figure 7:**
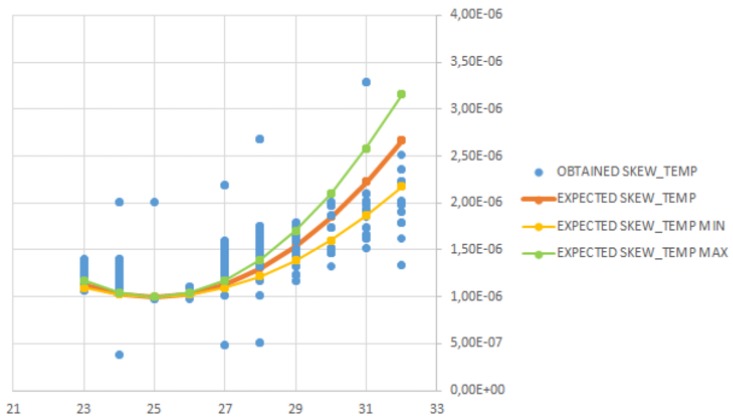
Obtained $skew_{\mathrm{temp}}$ compared to expected $skew_{\mathrm{temp}}$.

Figure 7 represents the expected $skew_{\mathrm{temp}}$ values taking into account that the temperature coefficient β=−0.034±0.006 has some deviation from the nominal value. All three possibilities have been incorporated to show the quadratic relationship (with the minimum, average and maximum value). The x-axis is the temperature, and the y-axis is the factor for $skew_{\mathrm{temp}}$ values. In the figure, as the remote node was set at a fixed temperature, the quadratic relationship of the $skew_{\mathrm{temp}}$ is more easily observed. The temperature has been rounded to $1^{\circ }$C in order to help the visualization. The range of values for the obtained $skew_{\mathrm{temp}}$ for each temperature is different, although there are no large deviations (<$10^{-6}$). These deviations are due to the use of the MAF, which provides an approximation to the LPF, and thus, the $skew_{\mathrm{cut}}$ is not as good as expected. In the figure, it can be observed that there is some gap between the expected $skew_{\mathrm{temp}}$ curve and the obtained $skew_{\mathrm{temp}}$ values. This difference is because of the MAF and the regression method used to compute the skew by FTSP. If the temperature remains stable for a period of time, some part of the amount assigned to $skew_{\mathrm{temp}}$ changes to $skew_{\mathrm{cut}}$, because the MAF include it in the computation. Besides, the FTSP regression method transfers some quantities to the offset value when the clock skew exceeds some value. Therefore, it is complex to observe a pure quadratic relationship of the $skew_{\mathrm{temp}}$ with respect to temperature. Nevertheless, this experiment clearly shows the quadratic behavior of the $skew_{\mathrm{temp}}$ measured in our experiments.

### 4.4. Conclusions about the Hypothesis

The above-mentioned results have empirically demonstrated that clock skew is: (i) a multiplicative combination of, at least, two components (as Vig [27] has suggested since a long time; however, he did not state the multiplicative combination behavior); (ii) the $skew_{\mathrm{cut}}$ shows a very stable behavior without any response to temperature variation; (iii) the $skew_{\mathrm{temp}}$ is highly correlated with any change in temperature; therefore, it includes all of the effects of temperature; and (iv) the proposed multiplication factor (correctionFactor) works instantly, helping the skew to reach the correct value faster than using $skew_{\mathrm{temp}}$.

## 5. Performance Evaluation

### 5.1. Experimental Setup

The improvement of wireless communications along with the reduced costs of electronic equipments have permitted the quick development of new low-cost wireless technologies. WSN are an example of merging between wireless communications and low-cost electronic equipment. Generally, a WSN is a group of low-cost and battery-powered motes. A WSN has a great range of applications, i.e., ambient monitoring, medical monitoring, security, among others. These applications are evolving in complexity, and for this reason, it is necessary to ensure the correct real-time performance through the use of wireless synchronization protocols.

As was described in the previous sections, this work is focused on the clock skew improvement in WSN by means of the separation of the main components involved. To test the hypothesis of Section 3, it is necessary to select a wireless protocol and a hardware platform. Following the testbed of previous works [9,15], the wireless communication protocol selected has been IEEE 802.15.4 [24] with the hardware platform TelosB. This hardware platform is equipped with a Texas Instruments 16-bit MSP430 [30] microcontroller with an internal digitally-controlled oscillator (DCO) working at 1-MHz and an external 32-kHz oscillator mainly used by peripherals, one of which is the Sensirion SHT11 temperature sensor (Sensirion, Staefa, Switzerland) [31]. The internal oscillator is synchronized with the external 32-kHz clock with an automatic adjustment of the internal frequency if there is a deviation between both clocks. In order to digitalize the temperature, a TinyOS module with real-time capability developed in [15] has been used.

#### 5.1.1. Time Synchronization Protocols’ Configuration

The new proposals (HF–FTSP and HF2–FTSP) are based on the previous work of Castillo et al. [15], as well as in the FTSP protocol of Maroti et al. [9]. For this reason, the execution testbed is the same used in the above-mentioned works. These parameters are listed in Table 6.

Besides, the variations introduced with several hops are not correlated with the proposed clock skew estimation method. That variation or jitter that appears in a multi-hop scenario is due to the communication lags between nodes, and this cannot be calculated with the clock skew estimation.

Moreover, in order to reduce the communication jitter, the time of the root node is excluded from the computation of the global time synchronization error. The root node is responsible of keeping the timing of the network, which produces the broadcast beacon messages that start the synchronization. However, there is a small delay between the instant when the root node begins the sending of the synchronization message and the actual instant when the message arrives at each node. That delay is commonly named communication jitter.

As the beacon message is broadcast to all of the nodes, it reaches at almost the same time all of the motes within a single-hop distance. Thus, if the root node is excluded from the computation of the differences, it is possible to minimize the communication jitter.

#### 5.1.2. Microseconds Accuracy in WSN Protocols

The goal is to synchronize all of the members of the network with the global network clock with the minimum offset. To reduce the uncertainties, timestamps have to be obtained in the lowest layer with respect to the physical layer. In this sense, Maróti and Ganeriwal [9,32] demonstrated that timestamping the packets in the MAC layer reduces the uncertainty times, and thus, errors are minimized.

The microsecond accuracy is obtained by using the internal DCO of the MSP430, which controls the timer where the local time is stored. TinyOS is responsible for the dynamic recalibration [33] of the clock of the DCO adjusting its working frequency according to the external 32-kHz oscillator frequency. Taking into account these statements, if DCO is running at 1 MHz (time clock is 1 μs), two consecutive pulses from the external crystal oscillators arrive at 1/32768 (≅31 μs). Thus, the frequency can be checked at least once each DCO modular period time, which is 32 DCO cycle times. Therefore, at most, the maximum average error is made for 31 DCO Clock cycles for each DCO clock cycle (31/32 μs, which is 0.96875 μs). However, that situation is not likely to be encounter, as that would mean that 31 times out of 32 the effective value was the incorrect one and only the one period left selected the proper frequency. Therefore, the MSP430F1611 [30] running TinyOS with a 32768 kHz oscillator is bound to a sub-microsecond average error (0.96875 μs) between two consecutive external crystal oscillator pulses.

#### 5.1.3. Topology

Following the configuration of [15], the topology of the experimental IEEE 802.15.4 network is composed of (1) a central mote that acts as the sink for obtaining the synchronization times from the nodes, (2) one mote broadcasting beacon frames for indicating the exact instant in which nodes have to transmit their synchronization information to the central mote and (3) thirteen motes flashed with FTSP, HF–FTSP and HF2–FTSP. Among the synchronized nodes, one of them is the root, which is responsible for keeping the global time of the network. For testing how temperature affects network synchronization, only three nodes (randomly selected) suffer the temperature variation inside a heated room controlled by a thermostat. The other nodes were under ambient temperature in a contiguous room separated by glass. Both rooms have been isolated in order to mitigate electromagnetic noise. The heat did not propagate uniformly, and therefore, some slight variations can be observed in the measured temperatures of the nodes at the same instants.

#### 5.1.4. Data Extraction

The performed tests have been executed during 3000 s. This time is enough to ensure the reliability of the extracted information. Once the information is extracted from the network, the sequence number of each packet has been used to group the information in order to obtain the results with MATLAB. By means of grouping the sequence numbers, it is possible to determine the average synchronization error for each temporal instant (Equations (15) and (16)). The average beacon time is the average global time of every node after a beacon has been broadcast by the root node, excluding in this computation the global time stored at the root node. This average beacon time tries to reduce the communication jitter, providing a consensus global time among the receiver nodes. Once the average error for each sequence number has been obtained, the global average synchronization error is determined using Equation (17).

(15) $$AvgBeacon=\frac{\sum_{i=1}^{nodes}\left(GlobalTime\left[i\right]\right)}{nodes}$$

(16) $$ErrorBeacon=\frac{\sum_{i=1}^{nodes}\left(AvgBeacon-GlobalTime\left[i\right]\right)}{nodes}$$

(17) $$ErrorGlobal=\frac{\sum_{j=1}^{beacons}\left(ErrorBeacon\left[j\right]\right)}{beacons}$$

where nodes is the number of nodes at the single-hop distance of the root node (excluding the root node) and beacons is the amount of beacons that has been broadcast within a certain elapsed time.

Other parameters with statistical relevance have been obtained: variance, typical deviation and maximum error.

## 6. Results

The obtained results show the performance of HF–FTSP and HF2–FTSP compared to FTSP. The experiment has been carried out in the south of Spain throughout several months over the year (in winter, spring and summer). This fact allowed three temperature-based case studies: low temperature ($9{\;}^{\circ }$C–$22{\;}^{\circ }$C), intermediate temperature ($22{\;}^{\circ }$C–$32{\;}^{\circ }$C) and high temperature ($22{\;}^{\circ }$C–$40{\;}^{\circ }$C).

The use of a correct BEACON_RATE value will determine the number of resynchronization periods and, therefore, the amount of energy used to synchronize the network. Based on previous works [29], the BEACON_RATE parameter has been set to 30 s to ensure: (1) a good synchronization rate that permits detecting if the temperature has changed to include it in the correction factor; and (2) for optimizing power consumption in the nodes by means of reducing the amount of sent packets. The number of elements in the linear regression has been set to three based on [15] experiments.

### 6.1. Low Temperature: $9{\;}^{\circ }$C–$22{\;}^{\circ }$C

Temperature changes modify the amount of tics in the oscillator of the mote. For this reason, it is important to test the homomorphic filtering approach under a great range of temperatures to demonstrate how it works under temperature variations. The first set of tests is the one performed between $22{\;}^{\circ }$C and $9{\;}^{\circ }$C. The tests start with a temperature of $22{\;}^{\circ }$C until the time instant 1000 s, where the temperature is decreased to $9{\;}^{\circ }$C.

**Figure 8:**
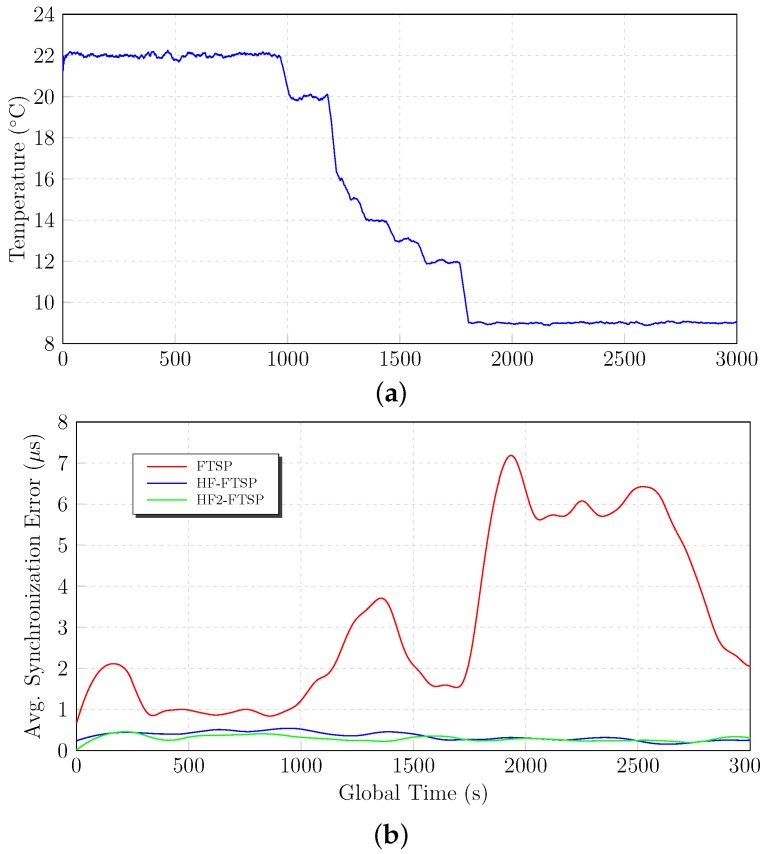
Low temperature results. (**a**) Temperature variation. (**b**) Average synchronization error.

Figure 8 shows the results for FTSP, HF–FTSP and HF2–FTSP in the temperature range $22{\;}^{\circ }$C–$9{\;}^{\circ }$C. The results until instant 1000 s are very similar between the above-mentioned approaches. Nevertheless, when the temperature change is induced at instant 1000 s, the differences between FTSP and the homomorphic-based solutions emerge. FTSP suffers drastically from the temperature effects when this changes, increasing its average synchronization error from 1.7 μs–12.2 μs at 1850 s. On the contrary, both HF–FTSP, as well as HF2–FTSP mitigate the temperature variations thanks to the separation of the components due to the cut of the crystal oscillator and the one due to temperature. In this case, the separation of the temperature component in the clock skew increases the performance of the synchronization. In the particular case of HF2–FTSP, the impact of the temperature change is negligible, as can be seen in Figure 8.

Attending to the final results of the low temperature experiments, Table 7 summarizes the average synchronization error, the standard deviation, the maximum value and the average error for 95% of the synchronization points. Attending to the average synchronization error, the homomorphic filtered approaches offer the better average synchronization error with 1.458 μs and 1.307 μs for HF–FTSP and HF2–FTSP, respectively. The influence of the temperature on FTSP is reflected in the final results, with an average synchronization error of 3.181 μs.

In order to summarize the behavior of both approaches in the test, Figure 8 shows the previous results through a local regression using weighted linear least squares and a second degree polynomial model. The behavior of the homomorphic filtering solution is clearly better than FTSP, demonstrating the capabilities of the separation of components to manage temperature variations.

### 6.2. Intermediate Temperature: $22{\;}^{\circ }$C–$32{\;}^{\circ }$C

The next set of tests is focused on the considered intermediate temperatures. The tests start fixing the temperature to $22{\;}^{\circ }$C during 1000 s. After the above-mentioned temporal instant, a temperature variation is provoked until reaching $32{\;}^{\circ }$C. Logically, this variation creates a skew in the oscillator frequency affecting the performance of the synchronization protocols.

During the first 1000 s of the test, the three approaches have similar results and behavior. All of them have an average synchronization error of about 1.6 μs. Nevertheless, when the temperature is changed at instant 1000 s, several differences appear between FTSP, HF–FTSP and HF2–FTSP. The homomorphic-filtered approaches have a similar behavior and results when the temperature reaches $32{\;}^{\circ }$C with an average synchronization error of 1.660 μs and 1.643 μs for HF–FTSP and HF2–FTSP, respectively. On the other hand, FTSP is clearly influenced by the temperature change from $22{\;}^{\circ }$C–$32{\;}^{\circ }$C, with an average synchronization error of 3.582 μs.

**Figure 9:**
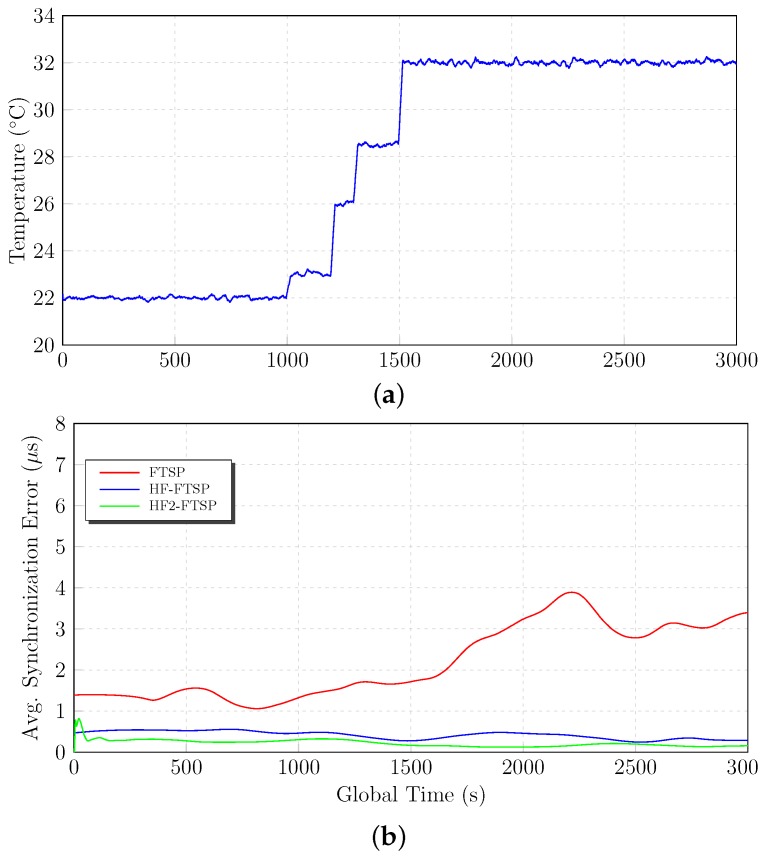
Medium temperature results. (**a**) Temperature variation. (**b**) Average synchronization error.

Analyzing the results summarized in Table 8 and Figure 9, a better performance of HF2–FTSP compared to the other approaches can be observed.

### 6.3. High Temperature: $22{\;}^{\circ }$C–$40{\;}^{\circ }$C

**Figure 10:**
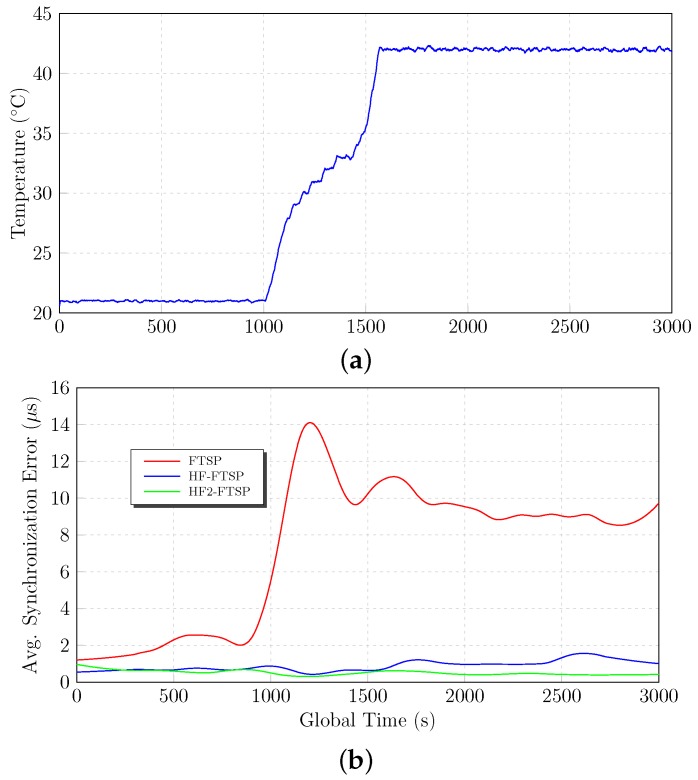
High temperature results. (**a**) Temperature variation. (**b**) Average synchronization error.

The following lines show the experimental results obtained with the parameters described in Section 5.1.1. As can be seen in Figure 10, the motes are affected by a temperature variation from $22{\;}^{\circ }$C–$40{\;}^{\circ }$C. During the fist 1000 s of the test, the homomorphic versions have an average synchronization error of 1.5 μs, while FTSP has an average synchronization error of 2 μs. When the temperature starts to change at instant 1000 s, several differences appear between the proposals.

FTSP suffers drastically from the consequences of the temperature change, getting a maximum average synchronization error of 20 μs at instant 1200 s. Behind that maximum value, the average synchronization error remains constant to an average synchronization error of about 9 μs until the end of the test. FTSP ends its test with an average synchronization error of 7.432 μs, as is summarized in Table 9.

The homomorphic proposals improve the results and the behavior of FTSP when the temperature is modified. HF–FTSP suffers slightly from the temperature variation at instant 1000 s, providing a maximum average synchronization error of 6 μs. On the other side, HF2–FTSP has a perfect behavior and results during all of the test, removing any influence caused by temperature in the result of average synchronization error.

As can be seen in Figure 10 and Table 9, both HF–FTSP and HF2–FTSP improve the results of FTSP for the considered high temperature range of the test. HF–FTSP gets an average synchronization error of 1.976 μs, while HF2–FTSP is about 1.298 μs. As was mentioned previously, FTSP suffers drastically the influence of the temperature with an average synchronization error of 7.432 μs.

## 7. Comparison with Other Previous Approaches

This section compares the new homomorphic filtered approaches with other temperature-based algorithms described in Section 1. The selected algorithms have been AT–FTSP, A2T–FTSP [15], TCTS [16], VHT [17], TACO [18] and EACS [19] because: (i) these synchronization protocols take into account temperature variations; (ii) are based on obtaining the clock skew of the nodes to improve the synchronization process; and (iii) are designed to be used in WSN. The performed tests for HF–FTSP and HF2–FTSP have been carried out under the same conditions of AT–FTSP and A2T–FTSP. Nevertheless, in the case of the TCTS, VHT, TACO and EACS algorithms, it is out of the scope of this work to readapt them to the experimental TelosB testbed described in this article due to the complexity of the task. However, the results published by their authors can be used for the sake of the comparison.

In the EACS scheme, Yang et al. [19] provided simulated and experimental results of their synchronization protocol. In the case of the simulation results, the authors state that the error is always below 2 ms for an 8000 s simulation test. On the other hand, for a test with Mica2 motes during 7200 s, the error was of 2 ms. According to the results of EACS, the frequency stability with an error of 8 ms in a period of 1000 s is 8 ppm.

In TCTS, Schmid et al. have performed simulated experiments in which, for an average beacon interval of 329 s, 95% of the errors lie within ±4 tics of a 32-kHz clock (122 μs). Analyzing these results, TCTS has a clock stability of 0.37 ppm for the above-mentioned beacon interval. Nevertheless, the authors state that for large resynchronization periods (4 ms of error in a 16-h period), the clock stability is less than 0.07 ppm. These authors improved much the precision of TCTS, proposing a new mechanism named VHT (Virtual High-resolution Time) [17]. With this service and TCTS, Schmid et al. have been able to provide high accuracy with low energy consumption under temperature variations. The authors have informed that a network of VHT-capable nodes running in a 14-h length experiment, with a 10-s resynchronization period provided a synchronization error that followed a Gaussian distribution of mean 0.125 μs and a standard deviation of 0.625 μs. These VHT nodes were equipped with two oscillators, one running at 32 kHz (for sleep intervals) and the other running at 8 MHz (for high-accuracy in processing periods). Therefore, the basic tic is the 8-MHz clock, which is 0.125 μs. Thus, VHT provides a mean synchronization error of one tic, and the standard deviation is five tics. This level of synchronization is excellent, although they achieve that with the addition of an 8-MHz oscillator, which increases the costs of the nodes.

Another protocol worth taking into account is TACO [18]. Xu et al. described a WSN system deployed in an intertidal zone and, thus, unable to keep the communication channels available all of the time. Besides, motes can be placed in dry places or in marine intertidal zones. Therefore, they suffered severely from temperature variations. These authors did not rely on a table of temperature and clock skew pairs, as the previous described approaches do. In TACO, they computed the correlation between temperature vs. clock skew by means of a model, predicting the clock skew for unseen temperatures. This behavior is very relevant for not-always available wireless channels. They provided real experiments that produced a maximum error of 1.71 ppm (591 ms in a four-day-length experiment), and 92% of the synchronization errors were below 1 ppm. They showed that their approach based on a model for predicting the clock skew for unseen temperatures behaved twice better than the EACS and TCTS approaches. Xu and Xu stated that their protocol suffered a clock drift of 57 ms after four days of simulated test execution with real temperature data, which is 0.165 ppm. Meanwhile, table-based schemes (EACS and TCTS) provided a clock drift of 168 ms in the same period of time, which is 0.486 ppm.

Another approach is the PulseSync protocol [34] by Lenzen et al. This approach shows a better behavior than FTSP in terms of average synchronization error and fewer synchronization messages along the network. Schmid et al. proposed TIRP [35], which provided similar results as PulseSync, although TIRP allows for asynchronous flooding. However, none of those protocols have been designed to compensate for temperature variations. Therefore, they have not been included in the comparison, although they are very interesting candidates to add the HF2 component, in order to make them temperature-aware.

In AT–FTSP [15], the authors carried out tests in a real-life scenario with TelosB motes. For a beacon interval of 30 s with temperature variations during 3000 s of test, AT–FTSP has a clock stability of 0.31 ppm for 95% of the errors. In the case of A2T–FTSP [15] and under the same conditions, the clock stability improved to 0.19 ppm. A 1-MHz timer compensated by the 32-kHz oscillator has been used in order to obtain microsecond accuracy. In Table 10, the compared results between AT–FTSP, A2T–FTSP, HF–FTSP and HF2–FTSP are shown. In general terms, the proposals based on homomorphic filtering of signals to separate the $skew_{\mathrm{cut}}$ of the $skew_{\mathrm{temp}}$ improve the AT/A2T proposals in terms of average error tics and clock stability.

These results show the convenience of separating the clock skew due to the cut of the crystal oscillator from the clock skew due to temperature effects. Furthermore, the new proposals keep the benefits of AT–FTSP and A2T–FTSP: (i) light in computational terms; (ii) based on obtaining the clock skew of the nodes to improve the synchronization process; and (iii) designed to be used in WSNs.

The results stated in Table 10 show the accuracy of the homomorphic filtering of signals applied to the clock skew estimation. The new proposals are designed to be used in low cost hardware platforms and then make it suitable to be used in any WSN platform. Besides, these new proposals open a new horizon of applications where it can be used as, for example, synchronization in cognitive radio wireless sensor networks [36,37,38,39,40] or Wireless Visual Sensor Networks (WVSNs) [41]. In the case of WVSNs, the cameras are distributed in a real environment; thus, they can be exposed to temperature variations. This decreases the performance of the synchronization and, as a consequence, affects the temporal ordering of the obtained images. In this sense, the use of synchronization protocols based on temperature variations, e.g. HF2–FTSP, is compulsory to ensure the correct reliability of WVSNs.

## 8. Conclusions

This work proposes two new methods to improve the clock estimation based on the concept of homomorphic filtering of signals for temperature variation scenarios in order to adjust the clock skew estimation in WSN synchronization protocols.

The new proposals use, in an innovative way, the concept of homomorphic filtering of signals applied to clock skew estimation in WSN. As a consequence, HF–FTSP and HF2–FTSP have been designed. These new proposals have the ability of separating the influence of the cut of the crystal oscillator and the influence of the temperature variations from the clock skew in order to obtain a better synchronization in the WSN.

Several tests have been carried out to determine the performance under different temperature scenarios. The obtained results show the convenience of separating the influence of the cut of the crystal and the one due to temperature effects to obtain a better clock skew estimation. The following paragraph summarizes the results of the test:

- The use of the homomorphic filtering of signals for the clock skew estimation minimizes the average synchronization error. The results show a clear improvement of the error compared to other similar WSN synchronization approaches.
- In the range of temperatures from $9{\;}^{\circ }$C–$22{\;}^{\circ }$C, HF–FTSP obtains an average synchronization error of 1.458 μs, and HF2–FTSP gets about 1.307 μs. These results supposes an improvement of 7% and 12% compared to AT–FTSP (1.558 μs) and A2T–FTSP (1.459 μs), respectively. If the comparison is with FTSP (3.181 μs), the improvement is about 118% for HF–FTSP and 143% in the case of HF2–FTSP.
- The intermediate temperature range (from $22{\;}^{\circ }$C–$32{\;}^{\circ }$C) makes HF–FTSP and HF2–FTSP improve, once again, the results compared to FTSP, AT–FTSP and A2T–FTSP. Nevertheless, the improvement is lower than the low temperature range. This behavior is logical because the temperature variations are near the nominal temperature of the crystal oscillator used in the experiment. HF–FTSP gets an average synchronization error of about 1.660 μs while HF2–FTSP gets 1.643 μs. These results suppose an improvement of 38% and 4% compared to AT–FTSP (2.302 μs) and A2T–FTSP (1.706 μs), respectively. When comparing with FTSP (3.582 μs), the percentages are 115% for HF–FTSP and 118% for HF2–FTSP.
- High temperatures ranges largely penalize the good behavior of the synchronization mechanisms. This is a consequence of the heat in the crystal oscillator and in the rest of electronics components of the mote. HF–FTSP (1.976 μs) has an improvement of 35% compared to AT–FTSP (2.685 μs), while HF2–FTSP (1.298 μs) improves A2T–FTSP (1.349 μs) about a 4%.

The main contributions of this article are below:

- It has been demonstrated that the clock skew is composed of, mainly, a couple of signals: the first one due to the cut of the crystal oscillator and the second one due to temperature effects. These signals are multiplicatively combined forming a non-linear system that can be separated in order to obtain both components.
- By using the homomorphic filtering of signals, it is possible to separate the above-mentioned multiplicative signals.
- Based on the concept of homomorphic filtering of signals, two new innovative clock skew estimation mechanisms have been developed: HF–FTSP and HF2–FTSP. Both proposals manage temperature variations by removing the temperature component in the combined signal and adding the new proposed temperature correction factor.
- The performed experimental results demonstrate that HF2–FTSP improves the FTSP, AT–FTSP, A2T–FTSP, EACS and TCTS synchronization protocols in terms of average synchronization errors.
- The results obtained make HF2–FTSP suitable to use in highly demanding synchronization accuracy applications.
- The new proposals allows reducing the economical costs of the hardware by using basics Crystal Oscillators (XO) instead of other models, such as Temperature-Compensated Crystal Oscillator (TCXO) or Oven-Controlled Crystal Oscillator (OCXO). These last models have the capability of mitigating temperature variations, but their economic costs are larger.
- The proposed methods can be applied in any synchronization protocol based on the clock skew concept.

## 9. Future Work

This work will be improved by testing the homomorphic filtered approaches in real-time-dependent wireless sensor network applications, such as WMSNs, data acquisition and data fusion, among others. Furthermore, it is interesting to test the new proposals in cognitive radio wireless sensor networks [36]. Cognitive radio networks enable efficient sharing of the radio spectrum. As Nieminen et al. [42] and Kailas et al. [43] state, synchronization schemes are necessary to improve the performance in cognitive radio wireless sensor networks. In that sense, it would be interesting to apply the homomorphic filtered synchronization approaches in a cognitive radio wireless sensor network in order to compare it with other similar proposals.

Finally, these mechanisms are to be designed in simulation platforms like OMNeT++ or MATLAB, which are the most commonly-used simulations tools for WSN. This will allow a quick testing scenario to obtain more relevant data about the performance and possible improvements of the proposals.

## Figures and Tables

**Table 1:** Terms and variables used in the mathematical model. HF, Homomorphic Filtered.

Variable	Description
*t*	Time.
$\vec{T}$	Vector of temperatures (log of temperatures).
skew	Skew obtained with any synchronization protocol.
$skew_{\mathrm{FTSP}}$	Skew obtained with FTSP.
$skew_{\mathrm{cut}}$	Skew due to the cut of the crystal oscillator.
$skew_{\mathrm{temp}}$	Skew due to temperature changes.
$skew_{\mathrm{avg}}$	Moving average of the obtained skews.
$skew_{\mathrm{HF}}-\mathrm{FTSP}$	Skew obtained with HF–FTSP.
$skew_{\mathrm{HF}}2-\mathrm{FTSP}$	Skew obtained with HF2–FTSP.
$\beta_{n}$	Temperature coefficient in node *n*.
$\beta_{r}$	Temperature coefficient in root node.
$T_{n}$	Temperature in node *n*.
$T_{r}$	Temperature in root node.
$T_{0}$	Nominal temperature ($25{\;}^{\circ }$C).
$\Delta T_{n}$	$T_{n}-T_{0}$.
$\Delta T_{r}$	$T_{r}-T_{0}$.

**Table 2:** Testbed parameters in the hypothesis demonstration experiments.

Parameter	Value
Beacon_Rate	10 s
Max_Entries	3 elements
Root_Timeout	5 periods
Ignore_Root_Msg	3 periods
Entry_Valid_Limit	3 elements
Entry_Throwout_Limit	500 μs

**Table 3:** Skew values obtained for Node #2. Skew values (columns 2→6) are in $10^{-6}$ notation.

T	${\mathrm{skew}}_{\mathrm{cut}}$	${\mathrm{skew}}_{\mathrm{temp}}$	$C_{\mathrm{factor}}$	${\mathrm{skew}}_{\mathrm{cut}}\cdot {\mathrm{skew}}_{\mathrm{temp}}$	${\mathrm{skew}}_{\mathrm{FTSP}}$	DiffSkew	Temp
500 s	40.5101	1.0146	1.0362	41.1015	41.1000	0.0015	$23.96{\;}^{\circ }$C
1000 s	41.1965	1.0222	1.0377	42.1110	42.1100	0.0000	$23.94{\;}^{\circ }$C
1500 s	40.9414	0.9306	1.0358	38.1000	38.1000	0.0000	$23.97{\;}^{\circ }$C
2000 s	41.8498	0.4442	1.0387	18.5895	18.5900	−0.0005	$23.93{\;}^{\circ }$C
2500 s	42.4464	1.0628	1.0001	45.1121	45.1100	0.0021	$24.94{\;}^{\circ }$C
3000 s	42.9368	0.9807	1.0001	42.1081	42.1100	−0.0019	$25.01{\;}^{\circ }$C

**Table 4:** Skew values obtained for Node #4. Skew values (Columns 2→6) are in $10^{-6}$ notation.

T	${\mathrm{skew}}_{\mathrm{cut}}$	${\mathrm{skew}}_{\mathrm{temp}}$	$C_{\mathrm{factor}}$	${\mathrm{skew}}_{\mathrm{cut}}\cdot {\mathrm{skew}}_{\mathrm{temp}}$	${\mathrm{skew}}_{\mathrm{FTSP}}$	Diff Skew	Temp
500 s	30.7593	1.0267	1.0375	31.5805	31.5800	−0.0006	$23.95{\;}^{\circ }$C
1000 s	31.7533	1.0260	1.1426	32.5788	32.5800	0.0010	$22.95{\;}^{\circ }$C
1500 s	33.1727	1.5564	2.2220	51.6299	51.6300	0.0001	$30.99{\;}^{\circ }$C
2000 s	38.3610	0.8436	1.1237	32.3613	32.3600	−0.0013	$26.90{\;}^{\circ }$C
2500 s	37.1540	1.0658	1.2910	39.5987	39.6000	0.0013	$27.92{\;}^{\circ }$C
3000 s	44.0121	1.9233	2.2191	84.6484	84.6500	0.0015	$30.98{\;}^{\circ }$C

**Table 5:** Quadratic relationship between $skew_{\mathrm{temp}}$ and TEMP.

Temperature	Expected ${\mathrm{skew}}_{\mathrm{temp}}$	Obtained ${\mathrm{skew}}_{\mathrm{temp}}$
$22.95{\;}^{\circ }$C	$1.1429\times 10^{6}$	$1.0260\times 10^{6}$
$23.95{\;}^{\circ }$C	$1.0375\times 10^{6}$	$1.0267\times 10^{6}$
$26.90{\;}^{\circ }$C	$1.1227\times 10^{6}$	$0.8436\times 10^{6}$
$27.92{\;}^{\circ }$C	$1.2899\times 10^{6}$	$1.0658\times 10^{6}$
$30.98{\;}^{\circ }$C	$2.2159\times 10^{6}$	$1.9233\times 10^{6}$
$30.99{\;}^{\circ }$C	$2.2199\times 10^{6}$	$1.5564\times 10^{6}$

**Table 6:** Testbed parameters.

Parameter	Value
Beacon_Rate	30 s
Max_Entries	3 elements
Root_Timeout	5 periods
Ignore_Root_Msg	3 periods
Entry_Valid_Limit	3 elements
Entry_Throwout_Limit	500 μs

**Table 7:** Results with temperature variation: $9{\;}^{\circ }$C–$22{\;}^{\circ }$C.

Parameter	FTSP	HF–FTSP	HF2–FTSP
Error	3.181 μs	1.458 μs	1.307 μs
Standard Deviation	5.829 μs	2.371 μs	2.179 μs
Maximum	50 μs	15.5 μs	23.7 μs
<95% Error	15.5 μs	8 μs	5.5 μs

**Table 8:** Results with temperature variation: $22{\;}^{\circ }$C–$32{\;}^{\circ }$C.

Parameter	FTSP	HF–FTSP	HF2–FTSP
Error	3.582 μs	1.660 μs	1.643 μs
Standard Deviation	6.156 μs	2.469 μs	2.392 μs
Maximum	87.500 μs	14.5 μs	17 μs
<95% Error	15.5 μs	7.5 μs	6.5 μs

**Table 9:** Results with temperature variation: $22{\;}^{\circ }$C–$40{\;}^{\circ }$C.

Parameter	FTSP	HF–FTSP	HF2–FTSP
Error	7.432 μs	1.976 μs	1.298 μs
Standard Deviation	8.757 μs	3.421 μs	1.911 μs
Maximum	44.5 μs	43 μs	20 μs
<95% Error	15.5 μs	9.5 μs	4.5 μs

**Table 10:** Comparative results. VHT, Virtual High-resolution Time.

Protocol	Avg. Synch.	Std. Dev.	Avg. Synch.	SD	Modified	Timer
	(tics)	(tics)	(error)	(error)	Hardware	Frequency
EACS	65.50 tics	N/A	2.000 ms	N/A	no	32 kHz
TCTS	4.00 tics	N/A	122.00 μs	N/A	no	32 kHz
VHT	1.00 tics	5.00 tics	0.125 μs	0.625 μs	yes	8 MHz
AT-FTSP	2.18 tics	3.70 tics	2.180 μs	3.700 μs	no	1 MHz
A2T–FTSP	1.50 tics	2.23 tics	1.500 μs	2.230 μs	no	1 MHz
HF–FTSP	1.69 tics	2.75 tics	1.690 μs	2.750 μs	no	1 MHz
HF2–FTSP	1.41 tics	2.16 tics	1.410 μs	2.160 μs	no	1 MHz

N/A: data not available data.
